# HealthMap: a cluster randomised trial of interactive health plans and self-management support to prevent coronary heart disease in people with HIV

**DOI:** 10.1186/s12879-016-1422-5

**Published:** 2016-03-05

**Authors:** Sarity Dodson, Karen M. Klassen, Karalyn McDonald, Tanya Millard, Richard H. Osborne, Malcolm W. Battersby, Christopher K. Fairley, Julie A. Simpson, Paula Lorgelly, Andrew Tonkin, Janine Roney, Sean Slavin, Jasminka Sterjovski, Margot Brereton, Sharon R. Lewin, Levinia Crooks, Jo Watson, Michael R. Kidd, Irith Williams, Julian H. Elliott

**Affiliations:** School of Health and Social Development, Deakin University, Geelong, Australia; Department of Infectious Diseases, Alfred Hospital and Monash University, Melbourne, Australia; Flinders Human Behaviour and Health Research Unit, Flinders University, Adelaide, Australia; Melbourne Sexual Health Centre and Department of Medicine, Central Clinical School, Monash University, Melbourne, Australia; Centre for Epidemiology and Biostatistics, Melbourne School of Population and Global Health, University of Melbourne, Melbourne, Australia; Centre for Health Economics, Monash University, Melbourne, Australia; Department of Epidemiology and Preventive Medicine, Monash University, Melbourne, Australia; Centre for Social Research in Health, University of New South Wales, Sydney, Australia; Department of Medicine, Dentistry and Health Sciences, University of Melbourne, Melbourne, Australia; Science and Engineering Faculty, Queensland University of Technology, Brisbane, Australia; Australasian Society for HIV Medicine, Sydney, Australia; Department of Public Health and Human Biosciences, La Trobe University, Melbourne, Australia; National Association of People with HIV Australia, Sydney, Australia; Faculty of Medicine, Nursing and Health Sciences, Flinders University, Adelaide, Australia

**Keywords:** Self-management, HIV, Cardiovascular disease, Health plans, Internet, Public health, Health services

## Abstract

**Background:**

The leading causes of morbidity and mortality for people in high-income countries living with HIV are now non-AIDS malignancies, cardiovascular disease and other non-communicable diseases associated with ageing. This protocol describes the trial of HealthMap, a model of care for people with HIV (PWHIV) that includes use of an interactive shared health record and self-management support. The aims of the HealthMap trial are to evaluate engagement of PWHIV and healthcare providers with the model, and its effectiveness for reducing coronary heart disease risk, enhancing self-management, and improving mental health and quality of life of PWHIV.

**Methods/Design:**

The study is a two-arm cluster randomised trial involving HIV clinical sites in several states in Australia. Doctors will be randomised to the HealthMap model (immediate arm) or to proceed with usual care (deferred arm). People with HIV whose doctors are randomised to the immediate arm receive 1) new opportunities to discuss their health status and goals with their HIV doctor using a HealthMap shared health record; 2) access to their own health record from home; 3) access to health coaching delivered by telephone and online; and 4) access to a peer moderated online group chat programme. Data will be collected from participating PWHIV (*n* = 710) at baseline, 6 months, and 12 months and from participating doctors (*n* = 60) at baseline and 12 months. The control arm will be offered the HealthMap intervention at the end of the trial. The primary study outcomes, measured at 12 months, are 1) 10-year risk of non-fatal acute myocardial infarction or coronary heart disease death as estimated by a Framingham Heart Study risk equation; and 2) Positive and Active Engagement in Life Scale from the Health Education Impact Questionnaire (heiQ).

**Discussion:**

The study will determine the viability and utility of a novel technology-supported model of care for maintaining the health and wellbeing of people with HIV. If shown to be effective, the HealthMap model may provide a generalisable, scalable and sustainable system for supporting the care needs of people with HIV, addressing issues of equity of access.

**Trial registration:**

Universal Trial Number (UTN) U111111506489; ClinicalTrial.gov Id NCT02178930 submitted 29 June 2014

## Background

### The changing health needs of people living with HIV

The population of people with HIV (PWHIV) is increasing and ageing. Rates of new infection are increasing in many high-income countries [1, 2], and the advent of effective combination antiretroviral therapy (cART) in 1996 resulted in a marked increase in life expectancy for PWHIV [3–5]. In the coming decades these factors are expected to result in a steady growth in HIV prevalence and a marked increase in the number of people with HIV living to older ages.

Despite increases in life expectancy, a substantial gap in life span persists between treated individuals with HIV and the general population [5–7]. In populations of PWHIV with high treatment coverage the key drivers of this gap in life expectancy and the majority of mortality and morbidity is now due to non-AIDS-related non-communicable diseases [8–10], particularly cardiovascular disease and non-AIDS malignancies [11–17] PWHIV are also at increased risk of other chronic medical conditions such as liver disease [15, 17], renal disease [18, 19], dementia [20], osteoporosis [21, 22] and depression [23, 24].

Cardiovascular risk is of particular concern. HIV infection appears to be independently associated with cardiovascular risk after adjustment for established risk factors [25]. This may be due to residual immune activation associated with HIV infection despite the achievement of virus suppression [26]. Some antiretroviral agents also appear to contribute to cardiovascular risk, either independently or in part mediated by an increased risk of dyslipidaemia and insulin resistance [27, 28]. However, the majority of excess cardiovascular risk in PWHIV is due to a high prevalence of established modifiable risk factors, particularly smoking and dyslipidaemia [29, 30]. Most studies of PWHIV in high-income countries indicate smoking prevalence of 30-50 %, usually more than double that in the general population [31]. Hypertension and diabetes are also important risk factors for coronary heart disease (CHD) both in HIV-infected and uninfected adults [32, 33].

### The increasing complexity of HIV care

The rising importance of prevention and management of chronic non-communicable comorbidities and persistently high levels of unmet psychosocial needs has resulted in increasing complexity of HIV care [34]. In response, health care services need to provide chronic disease prevention and management and mental health care alongside specific treatment for HIV itself [35, 36].

Data reporting the quality of care provided to patients with HIV is sparse, but suggests an evidence-practice gap in critical aspects of contemporary HIV care. In Australia, an audit of 500 patients initiating cART at four hospital and primary care sites suggested the concordance between practice and guideline recommendations in these sites was generally high (>70 %) for HIV treatment activities, but low (<50 %) for activities in relation to chronic co-morbidities [37, 38].

Persistent policy aspirations for HIV care to be provided from within a chronic care framework with an emphasis on self-management [39] are yet to be broadly realised. Only a few HIV-specific self-management support programmes have been developed [40–43] and linkages between these programmes and HIV treatment services are rarely established, compounding fragmentation of care.

### Responding to the increasing complexity of HIV care: the HealthMap model

The HealthMap model has been developed in response to the gap between policy and the implementation of critical aspects of care for PWHIV in practice. It links the usual interactions of the existing care team to a set of self-management support opportunities with the aim of reducing the risk of coronary heart disease and improving the psychosocial wellbeing of people with HIV. The model includes 1) new opportunities for people with HIV to discuss their health status and goals with their doctor using the HealthMap shared health record; 2) access for people with HIV to their own health record and contextual health information from home; 3) self-management support delivered by telephone and online; and 4) access to an online peer moderated group chat programme.

It is intended that, by utilising within healthcare consultations, an interactive software platform that provides convenient access to patient health information, doctors and their patients will more frequently and more easily engage in conversations about the patient’s health status and broader health priorities. The addition of self-management support services integrated with patients’ usual primary health care is intended to supplement health promotional activities in the health clinic and support patients’ ability to progress towards their health-related goals.

### Evaluation of the HealthMap model

This paper describes the protocol of a cluster randomised controlled trial of the HealthMap model. These are specific aims of the HealthMap trial:

#### Aim 1

Evaluate the effect of an interactive shared health record and integrated self-management support on coronary heart disease risk, disease self-management, mental health and quality of life of PWHIV.

#### Aim 2

Evaluate patient and health care provider engagement with, and acceptance of, an interactive shared health record and integrated self-management support.

#### Aim 3

Evaluate the cost-effectiveness of an interactive shared health record and integrated self-management support.

#### Hypothesis

The use of interactive, within-consultation decision supports and integrated self-management support will result in improved cardiovascular risk and self-management behaviours in people with HIV, and will be acceptable and cost-effective.

## Methods

### Study design

**Fig. 1:**
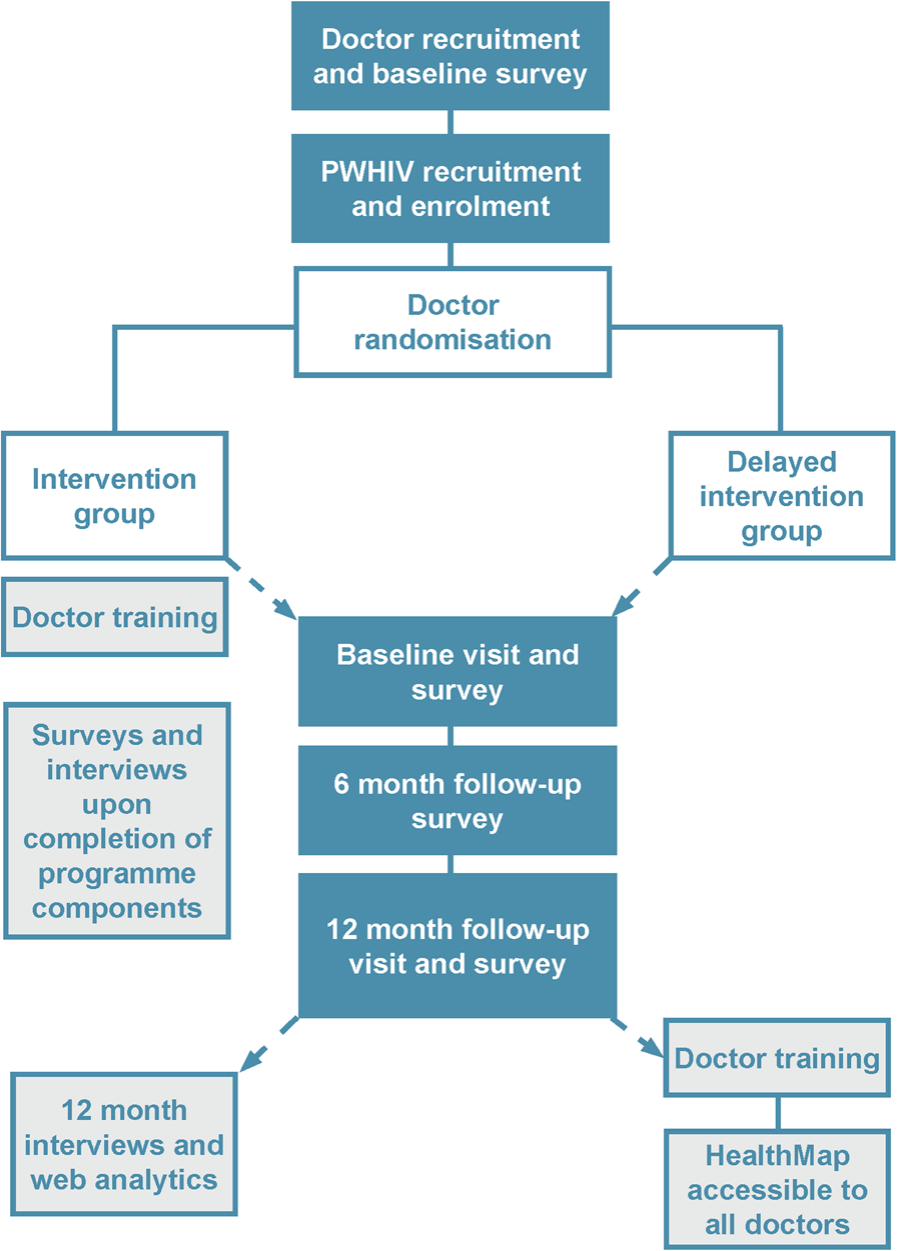
Flow chart of the study processes

The study is a two-arm cluster randomised controlled trial of the HIV HealthMap model, with clustering applied at the level of the doctor. Doctors will be randomised to the HealthMap model (immediate arm) or proceed with usual care (deferred arm). Patients with HIV will be allocated to the study arm to which their usual HIV doctor has been randomised. Doctors and patients in the deferred arm will continue with usual care until the end of the trial, after which they will be able to access the HealthMap model. Data will be collected from PWHIV participants (*n* = 710) at baseline, 6 months, and 12 months and from doctor participants (*n* = 60) at baseline and at 12 months. A flow chart of the study is presented in Fig. 1.

### Study populations

Doctors are eligible to participate if 1) their clinic uses electronic patient management systems capable of integrating with HealthMap systems to enable capture of pathology data; and 2) they provide care to a minimum of five PWHIV. Patients are eligible to participate if 1) they are aged 30 or over; 2) they receive primary HIV care and general care from a participating doctor and are likely to continue to do so for 12 months; 3) they do not have diagnosed cardiovascular disease (i.e., coronary health disease, stroke, transient ischaemic attack, or peripheral arterial disease); 4) they have not previously participated in an HIV-specific self-management or coaching programme; and 5) they are willing and able to provide written informed consent.

### Study outcomes

All study outcomes are to be measured at the 12 month follow-up visit. Secondary outcomes measured by an online survey will also be measured at 6 months. The primary study outcomes are 1) 10-year risk of non-fatal acute myocardial infarction or coronary heart disease death as estimated by a Framingham Heart Study risk equation [44]; and 2) their Positive and Active Engagement in Life Scale score (one of eight scales of the Health Education Impact Questionnaire heiQ) [45]. The secondary study outcomes are as follows:

1. Cardiovascular risk relative to age as measured by the Joint European Taskforce relative risk tables [46]
2. Cardiovascular risks estimated by an HIV-specific risk score [28]
3. Smoking status as measured by self-report and verified by urinary cotinine in individuals who report smoking at baseline and not smoking for at least 1 month prior to the 12 month visit [47, 48]
4. Fasting total cholesterol and total cholesterol to HDL ratio (measured by serum assay)
5. Systolic blood pressure (measured using a sphygmomanometer)
6. Body mass index (weight in kilograms divided by height in metres squared) and waist circumference (measured at the level of the umbilicus using a flexible steel measuring tape)
7. Proportion of patients achieving Australian cardiovascular risk factor management targets [49]
8. Health-related quality of life as measured by the AQoL-4D instrument [50]
9. Mental health status as measured by the DASS-21 instrument [51]
10. Self-monitoring and insight as measured by the heiQ [45]
11. Constructive attitudes and approaches as measured by the heiQ [45]
12. Social integration and support as measured by the heiQ [45]
13. HIV stigma coping strategies as measured by Herek and colleagues HIV stigma scale [52]
14. Proportion of patients with HIV virus suppression (below the lower limit of detection of the assay used)
15. Proportion of patients achieving HIV quality of care measures [53]

### Study setting

The study will be conducted in HIV clinical sites across Australia, including general practice clinics, public hospitals and sexual health centres.

### Recruitment procedures

Doctors will be identified using existing professional networks and national clinic data, and invited to participate in the study. Eligible doctors, and research coordinators will provide written consent, receive training in the conduct of the study, and participate in the development of site-specific study procedures. Participating doctors and research coordinators will approach potential patient participants sequentially, describe the study, and ask them to provide consent. The study will be promoted through community and online channels.

### Randomisation procedures

Cluster randomisation will be performed at the level of treating doctor by a statistician, after stratification for doctor type (general practitioner versus physician) and number of enrolled patients (above and below median). Doctors will be randomly assigned 1:1 to either immediate (intervention arm) or deferred (control arm) access to the intervention package. Random allocation will be performed using the ‘ralloc’ program in the statistical package Stata 13 [54].

### Intervention Training

Doctors randomised to the intervention arm will receive individual training regarding the use and integration of the HealthMap platform within their clinical consultations.

### The HealthMap model

**Fig. 2:**
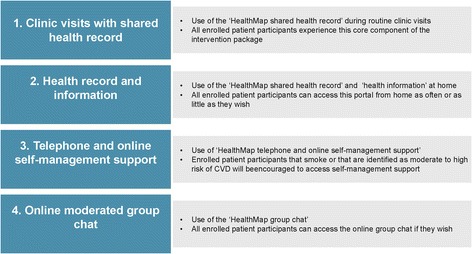
Intervention components – the HealthMap model

PWHIV in the immediate access arm of the study will experience 1) new opportunities during their routine clinic visits to discuss their health status and goals with their doctor using the HealthMap shared health record; 2) access to their own health record and contextual health information from home; 3) access to health coaching delivered by telephone and online using the *SteppingUp* health coaching programme [55] and 4) access to an online peer moderated group chat programme (Fig. 2).

#### Component 1: Clinic visits using the HealthMap shared health record

Participants will visit their HIV doctor as per their usual schedule, typically every three to 6 months, to review blood test results and obtain a prescription for cART and other medications. At these visits, doctors and their patients will use the HealthMap shared health record as a tool for facilitating discussion about recent laboratory test results and identifying health issues and areas where the patient is interested in making changes. Specifically, doctors will use the shared health record to:

1. Present recent laboratory results
2. Discuss the implications of these results
3. Agree upon and document health priorities with the patient
4. Discuss strategies to address these health priorities
5. Refer patients to self-management support coaching
6. Review and track progress over time, making adjustments to priorities and strategies as needed

**Fig. 3:**
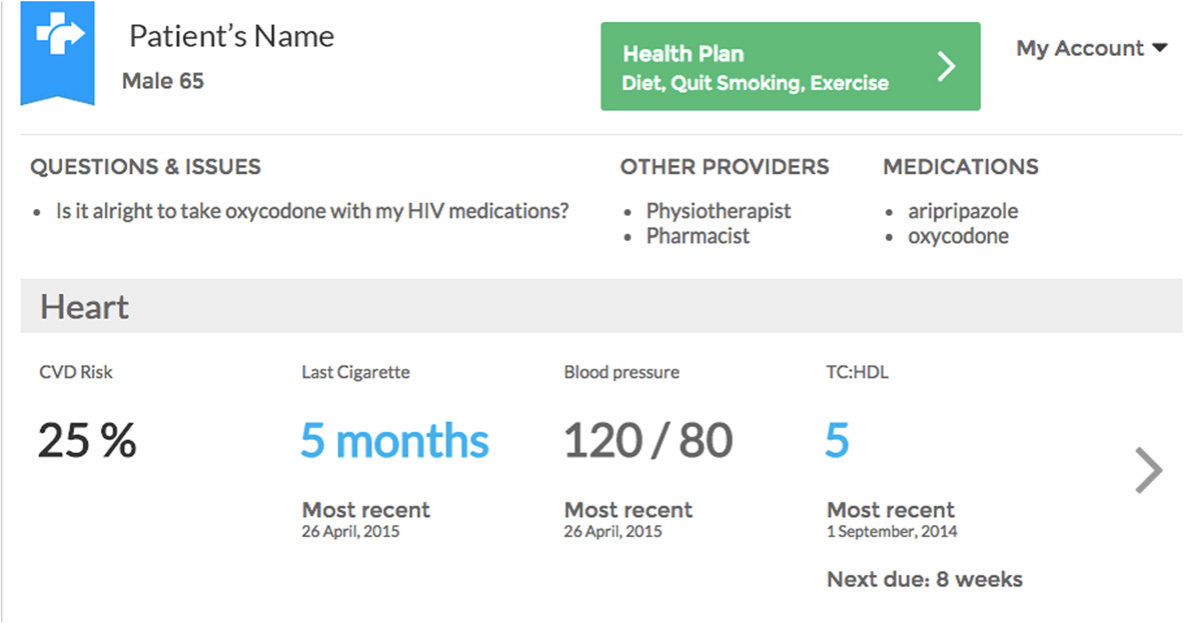
A screenshot of the doctor’s view of patient information

Linkages between the ‘HealthMap health-planning pages’, the ‘HealthMap PWHIV information and results pages, and the ‘coach pages’ will allow common views of key information between patients, coaches and providers. The HealthMap website is accessed via a secure web browser. A screenshot of the doctor’s view of patient information is shown in Fig. 3.

#### Component 2. Access to the HealthMap shared health record and information from home

**Fig. 4:**
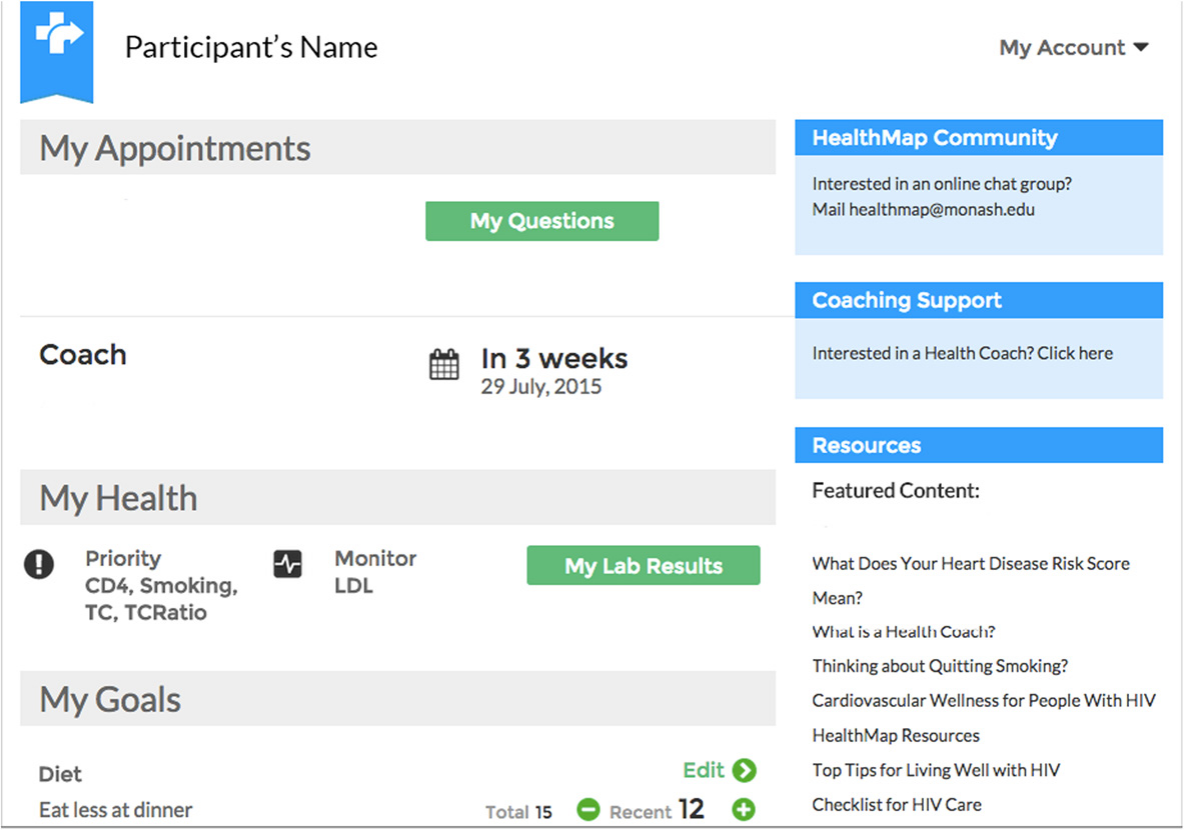
A screenshot of the patient dashboard

All enrolled patient participants will be encouraged to use the HealthMap shared health record outside of clinic visits. The shared health record provides PWHIV with access to their laboratory results captured from their clinic health record, health priorities identified with their doctor, and action plans to make health changes. In addition, health information and links to additional resources relevant to each individual’s health profile are presented. A screenshot of the patient dashboard is shown in Fig. 4. Specifically, patient participants will be able to:

- Review the laboratory results shown to them during their clinic visit
- Access information describing their health priorities, goals and planned actions
- Create or update action plans to address health goals
- Document progress towards their health goals
- Register interest in the health coaching programme if they currently smoke or are at a high CHD risk
- Register interest in the online peer moderated group chat
- View details of upcoming coaching appointments (if applicable)
- Send messages to their health coach
- Document areas of concern they wish to discuss during their next clinic visit
- Access patient education resources

#### Component 3. Telephone and online health coaching using the *SteppingUp* health coaching programme

Patient participants identified as smokers or at moderate-to-high risk of cardiovascular disease (>10 % risk of cardiovascular disease over the following 5 years) will be encouraged by their regular doctor and other clinic staff to participate in phone and online self-management support.

**Fig. 5:**
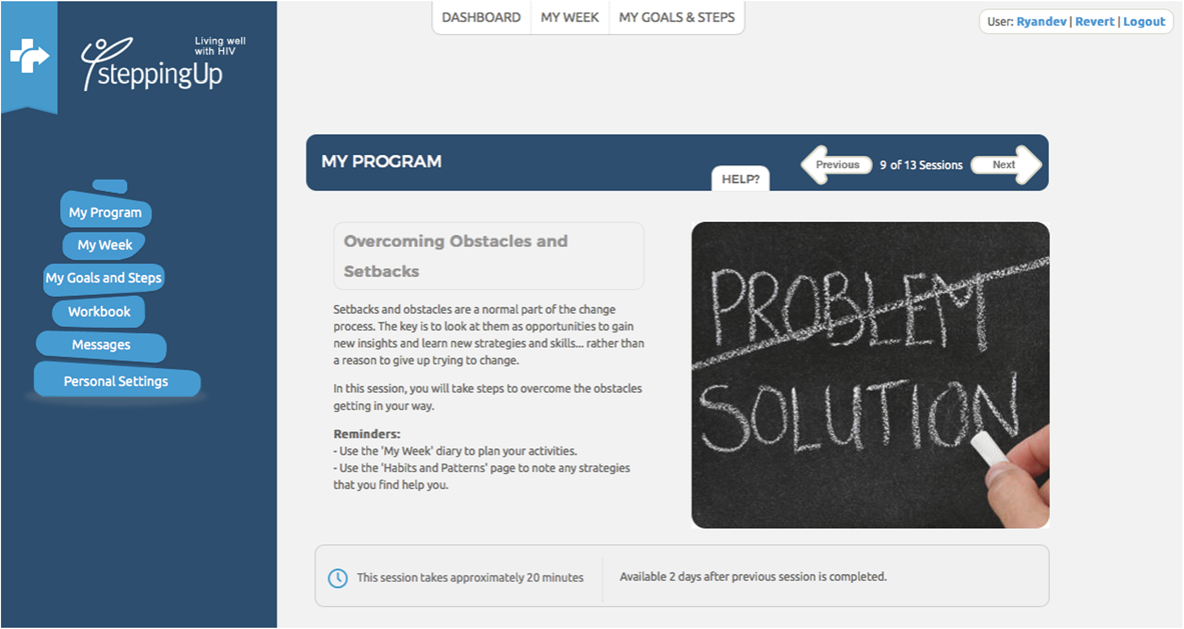
A screenshot of the *SteppingUp* online platform

The self-management programme will be delivered by telephone or via a secure online portal with email support from a coach. Patients enrolled into the coaching programme will have an initial telephone assessment that seeks to identify health knowledge and behaviours, health priorities and barriers to self-management. The Flinders Program assessment tools form the basis of this assessment process [56] The interview will also involve a collaborative goal setting process. Where a patient opts to engage with the coach supported online programme, they will be assigned a series of tailored online learning modules to work through at their own pace using the *SteppingUp* online platform [55]. The assigned online programme will be matched to the patient’s identified treatment goal. A screenshot of the *SteppingUp* online platform is shown in Fig. 5.

Patients will have access to the programme from between 4 and 12 weeks, depending on their needs and circumstances. Assessment and progress information will be recorded by the coach in the HealthMap shared health record and remain accessible to the coach and treating doctor.

#### Component 4. Online group chat

All enrolled patient participants will be invited to take part in a peer moderated online chat group. Invitations will come from clinic staff, as well as via prompts delivered in the HealthMap shared health record, and by email and SMS. Patients will be able to register their interest and will be assigned to a group suited their specified gender or sexual identity preference. An experienced peer moderator will manage enrolments, assign patients to appropriate groups, and make contact with patients with commencement details. Each chat group will involve a two-hour moderated online chat session, once per week for 6 weeks. Up to 10 participants will be enrolled in each group. Sessions will be based on the Positive Outlook Program [57] and focus on pre-assigned topics including issues of disclosure of HIV status and negotiating intimate relationships.

### Data collection from participating patients

#### Baseline visit

Following randomisation, recruited patient participants will be asked to attend a baseline study visit. Participants will be asked to complete an online survey prior to the study visit or during the study visit. The survey includes measures of 1) health-related quality of life, 2) chronic disease self-management, 3) mental health, 4) health behaviours (smoking and physical activity), and 5) HIV stigma. During the study visit, clinical and demographic data will be collected by site staff and blood pressure, weight, height and waist circumference will be measured.

#### Six month follow-up

Participating PWHIV will be emailed or sent a text message by HealthMap research staff asking them to complete the online survey. The window for this data collection will be 6 to 8 months.

#### Upon completion of programme components

PWHIV who participate in the telephone and online self-management support, or the online moderated group chat components will be asked to complete online surveys at completion of these activities. These surveys will enquire about patient’s satisfaction with these components, and their perception of the degree of benefit gained from their engagement with these intervention components.

In addition to these surveys, a minimum of ten participants of the self-management support component, and a minimum of ten of the online group chat component who have previously consented to be contacted for a telephone interview will be selected to take part in a semi-structured interview. Participants exposed to both components will not be approached for interview. The interviews will focus on four key areas: 1) current healthcare management practices; 2) communication and joint decision making with doctors; 3) engagement and adherence to self-defined goals and other recommended healthcare activities; and 4) sources of information and support. Participants’ experiences with the HealthMap platform, self-management support and online chat groups will also be explored.

#### Twelve month follow-up

Participating PWHIV will be asked to attend a study visit 10 to 14 months following their baseline visit. During this visit, clinical data collected at baseline will be repeated. Additionally, a urine sample will be collected from participants who reported smoking at baseline and smoking abstinence for at least 1 month prior to the 12 month visit. Patients will be asked to complete the online survey prior to the study visit or during the study visit.

A minimum of ten participants will also be selected to take part in a semi-structured interview. Participants taking part in the optional telephone and online self-management support component or the online moderated group chat component will not be approached. These interviews will follow the same structure as those conducted with participants of the additional programme components.

### Data collection from participating service providers

#### Baseline

Participating doctors will complete a survey at baseline that includes details of their HIV experience and workload and current chronic disease management practice.

#### Twelve months from baseline

All intervention arm doctors, coaches and peer chat moderators will be asked to complete a short online survey about their experiences of the HealthMap model. Coaches, peer chat moderators and a minimum of ten intervention doctors will also be invited to participate in semi-structured interviews. These interviews will focus on their general experience of the HealthMap model, perceptions of effectiveness, clinical burden, and implementation issues associated with delivery of each HealthMap intervention component.

### Data collection from the online platforms

The following data will be extracted from the online platforms (i.e., the main HealthMap electronic shared health record, the moderated group chat, and the self-management support): number, frequency and distribution of participant and doctor logins, individual webpage views, participation and posts in the online groups, and interactions between health coaches and patient participants.

### Sample size calculation

For the co-primary outcome of CHD risk, we used the primary dataset of STEAL (*n* = 280; J. Amin, personal communication), a multi-site Australian HIV clinical trial [58] to estimate a geometric mean (due to skewed distribution) Framingham 10 year coronary heart disease risk [44] of 6.3 % units with standard deviation of 0.70 (log_e_ %units) and an intra-class correlation coefficient of 0.058. We then evaluated the Framingham 10 year coronary heart disease after we enrolled 563 participants. The geometric mean (due to skewed distribution) Framingham 10 year coronary heart disease risk was 6.9 % units with standard deviation of 0.72 (log_e_ %units) and an intra-class correlation coefficient of 0.09 (to account for the differences in CHD risk between the doctors). We estimated a minimum clinically important relative reduction of 24 % in Framingham risk based on similar effect sizes in previous self management [59] and cardiovascular risk management studies [60]. We conservatively estimated loss to follow up to the 12 month primary outcome as 15 %, five times the 96 week loss to follow up in the STEAL study [58]. Using clustersampsi in Stata (Version 12, College Station, TX, USA) and using a coefficient of variation of 1 (to account for the variation in the number of patients enrolled per doctor), we calculated that to provide 80 % power at a 5 % significance level (two-sided) we will require 60 doctors and a total sample size of 710 patients (355 per arm and an average 12 patients per doctor).

For the co-primary outcome of the Positive and Active Engagement in Life (PAEL), we used the primary dataset from an archived dataset held within Deakin University’s heiQ database. The most comparable intervention was selected from among hundreds of known interventions to match the likely impact of HealthMap. The dataset included *n* = 212 individuals who had an absolute mean gain in PAEL of 0.35 units with a standard deviation of 0.46 in the intervention group. Using clustersampsi in Stata (Version 12, College Station, TX, USA), a coefficient of variation of 1 for size of cluster and an intraclass correlation coefficient of 0.09, we calculated that to provide 80 % power at a 5 % significance level (two-sided) we will require 60 doctors and a total sample size of 142 patients (71 per arm and an average 2 patients per doctor). This sample size allows for a 15 % loss to follow up rate.

### Data and safety monitoring board

A Data and Safety Monitoring Board (DSMB) and Protocol Steering Committee (PSC) will be established. The DSMB will consist of a statistician, an experienced HIV clinician and a cardiologist, without previous involvement in the trial. The DSMB will oversee the baseline coronary heart risk in study participants and provide advice as to whether the projected power of the study is likely to be achieved. Interim summary coronary heart risk data collected at the time of enrolment will be submitted to the DSMB after 25, 50 and 75 % of participants have been enrolled.

### Ongoing reporting

Doctors, other study site staff, coaches and moderators will record issues arising during the study in an issues log and report significant issues to the HealthMap study team within 24 h. The HealthMap study team will report all significant incidents to Human Research Ethics Committees within 72 h of the incident. All incidents will also be reported to the Protocol Steering Committee.

### Data analysis

All statistical analyses will be conducted using Stata 13 [54] according to the pre-specified plan below. All of the primary and secondary outcomes are measured at the individual level and therefore we will use Generalised Estimating Equations (GEEs) to allow for the clustering due to randomisation at the doctor level.

### Examination of primary outcomes

The distribution of Framingham risk scores and PAEL are typically skewed, so scores will be log_e_ transformed where necessary. An estimate and 95 % confidence interval of the relative difference in geometric mean (at 12 months) between the intervention and control groups will be derived using linear regression, with adjustment for baseline scores and fitting using GEEs to allow for clustering by doctor.

### Examination of secondary outcomes

For the continuous secondary outcomes measured at the six and/or 12 month visit: coronary heart risk (relative to age and as estimated by HIV-specific risk score); fasting total cholesterol and total cholesterol: HDL ratio; systolic blood pressure; body mass index and waist circumference; and quality of life and mental health status scores; an estimate of the absolute mean difference (or relative difference in geometric means for outcomes with a lognormal distribution) and 95 % confidence interval between the intervention and control groups will be derived by linear regression using GEE (to account for clustering by doctor) with adjustment for, where applicable, baseline measures of the secondary outcome.

For the binary secondary outcomes measured at the 12 month visit: smoking status (smoker versus non-smoker); achievement of Australian cardiovascular risk factor management targets (yes versus no); HIV virus suppression (yes versus no); and achievement of HIV quality of care measures (yes versus no); an estimate of the Odds Ratio (OR) and 95 % confidence interval for the intervention compared to the usual care will be derived by logistic regression modelling using GEEs, to allow for clustering by doctor. To identify latent HR-QoL and self-management profiles at baseline, Latent Profile Analysis will be conducted using MPlus software package (ref). Associations between latent profiles at baseline and both demographic characteristics and change on binary secondary outcomes will be identified. This approach will allow intervention responsiveness to be explored within the context of baseline self-management practices and functioning.

### Economic evaluation

The health economic analysis will estimate the cost-effectiveness of the intervention relative to standard care, primarily in terms of reducing coronary heart disease risk, but also in terms of improving life expectancy and quality of life. The economic evaluation will be conducted alongside the clinical trial and a Markov model will be employed to extrapolate beyond the trial period to estimate lifetime cost effectiveness. Resource use and costs associated with providing standard care and the management plans will be collected and compared. Resource use will be valued using standard reference pricing and unit costs, primarily by using the Medicare Benefits Scheme and the Pharmaceutical Benefits Scheme, the mechanisms by which the Australian government funds specific health care. Total costs will be estimated at an individual level, and average costs will be compared between the two study arms.

The relationship between costs and outcomes will be considered and incremental cost effectiveness ratios (ICERs) estimated, for the trial period and over a lifetime. The within trial analysis will use absolute change in coronary heart disease risk and a generic quality of life measure, the AQoL-4D [50] to estimate quality adjusted life years (QALYs). Cost per reduction in coronary heart risk and cost per QALY gained will be estimated. Sensitivity analyses will be undertaken, using non-parametric bootstrapping, to understand the uncertainty surrounding the ICER estimates.

To understand the long-term cost effectiveness of each intervention it will be necessary to extrapolate and model future events. Estimated risk reductions for each patient in the intervention and control groups will be extrapolated to life years saved using a Markov type model which converts risk reductions into events avoided (using published evidence) and then into life years saved over the patient’s lifetime. The trial AQoL and QALYs estimates will supplement estimates from the literature of the utility reduction associated with various coronary heart events, and the Markov model will estimate the QALYs gained over a lifetime. These lifetime estimates of effect will be combined with costs to generate a measure of the cost per life year saved and cost per QALY gained associated with the intervention, thereby determining the long-term cost effectiveness of interactive self-care plans and self-management support for PWHIV.

### Management of missing data

The distribution of the baseline characteristics and outcome measures recorded at baseline will be presented for those with and without data available at the 12 month follow-up, and compared using multivariable logistic regression where the outcome variable is data available at 12 month follow-up visit (yes versus no). If the proportion of missing data at the 12 month visit is <10 %, then a complete-case analysis will be performed (i.e., those participants with missing data are excluded from the statistical analysis). Otherwise, two statistical approaches will be considered for handling the missing data: a complete-case analysis as the primary analysis and the application of multiple imputation as a sensitivity analysis [61]. For the imputation stage of multiple imputation, the imputation model will be developed based on the main analysis model, the findings of the multivariable logistic regression above of the baseline covariates for consideration of auxiliary variables, and the data available from the 6 month online survey. Reporting will adhere to the guidelines suggested by Sterne JAC et al. [62].

### Management of qualitative data

Qualitative interview data will be transcribed verbatim and identifying details anonymised. Data will be entered into NVivo 10 (QSR International Pty Ltd, 2012) and coded for a structured thematic analysis. Demographic and health status data from patients will be used to identify patterns of experience.

### Ethical approval

This study has received ethical approval from Alfred Health Human Ethics Committee (520/13), the Royal Australian College of General Practitioners (NREEC 13–015), Monash University Human Research Ethics Committee (CF14/925–2014000367) and the Australian Department of Health Ethics Committee (SF4060527).

### Trial registration

The trial is registered at clinicaltrials.gov (NCT02178930) and anzctr.org.au (U1111-1150-6489).

## Discussion

The HealthMap model of care aims to encourage people with HIV and their doctors to engage more actively in addressing the risk factors that contribute to the high coronary heart risk observed in PWHIV. This cluster randomised control study seeks to determine the effectiveness of the HealthMap model of care in reducing coronary health disease risk, improving self-management, mental health and quality of life of PWHIV. It also seeks to evaluate provider and patient engagement with the model’s components, and the acceptability and cost-effectiveness of this approach to care.

The study will contribute to our understanding of the feasibility and effectiveness of novel chronic care approaches to the management of HIV and the benefit and impact of introducing interactive software components into clinical consultations for enhanced chronic disease management. If shown to be effective and cost-effective, the HealthMap model of care may provide a generalisable, scalable and sustainable system for improving the long term health of people with HIV, assuring the quality and safety of HIV care, supporting healthcare worker engagement in HIV care provision, and improving equity of access to high quality HIV care programmes.

## Abbreviations

cART: combination antiretroviral therapy
CHD: coronary heart disease
DSMB: data and safety monitoring board
heiQ: Health Education Impact Questionnaire
OR: odds ratio
PSC: Protocol Steering Committee
PWHIV: people with HIV
